# Hybrid Complexes of Photosensitizers with Luminescent Nanoparticles: Design of the Structure

**DOI:** 10.32607/actanaturae.11379

**Published:** 2021

**Authors:** D. A. Gvozdev, E. G. Maksimov, M. G. Strakhovskaya, V. Z. Pashchenko, A. B. Rubin

**Affiliations:** M.V. Lomonosov Moscow State University, Department of Biology, Moscow, 119991 Russia

**Keywords:** FRET, photosensitizer, luminescent nanoparticle, photodynamic therapy

## Abstract

Increasing the efficiency of the photodynamic action of the dyes used in photodynamic therapy is crucial in the field of modern biomedicine. There are two main approaches used to increase the efficiency of photosensitizers. The first one is targeted delivery to the object of photodynamic action, while the second one is increasing the absorption capacity of the molecule. Both approaches can be implemented by producing dye–nanoparticle conjugates. In this review, we focus on the features of the latter approach, when nanoparticles act as a light-harvesting agent and nonradiatively transfer the electronic excitation energy to a photosensitizer molecule. We will consider the hybrid photosensitizer–quantum dot complexes with energy transfer occurring according to the inductive-resonance mechanism as an example. The principle consisting in optimizing the design of hybrid complexes is proposed after an analysis of the published data; the parameters affecting the efficiency of energy transfer and the generation of reactive oxygen species in such systems are described.

## INTRODUCTION

Theranostics, which combines photodynamic therapy (PDT) and fluorescence diagnostics, is a promising field in modern medicine that uses light to detect and eliminate tumors, other unwanted structures, as well as the foci of microbial and fungal infections of the skin and mucous membrane [1, 2]. Photodynamic reactions are carried out by dye molecules capable of absorbing a quantum of light and passing into a long-lived triplet state. During its deactivation, a dye molecule produces reactive oxygen species (ROSs) and free radicals. ROSs possess high oxidative activity and can be used to disrupt the functionality of individual biomolecules and the vital activity of whole cells. Such dyes are called photosensitizers (PSs). These are typically complex heterocyclic compounds with a number of absorption bands in the visible spectral range. A substantive search for highly effective PSs that can be used within the phenomenon of photosensitization for the treatment of cancer and infectious diseases is currently underway. A number of synthetic PSs are already successfully being used in clinical practice to fight certain types of cancer, in dentistry, etc. [3].

One of the key criteria in choosing dyes for PDT is the significant absorption capacity of PS in the red and near-infrared spectral regions, since light penetration depth into biological tissues is considered to be the greatest in this range. Modifying the structure of a PS molecule might be an inefficient way to meet this criterion; therefore, it might be required to use additional light collectors. Having absorbed light of the required spectral range, the light collector will transfer energy to the PS and, thereby, enhance its photodynamic effect.

The main mechanism of energy transfer in such hybrid complexes (HCs) is considered to be the nonradiative one (Forster resonant energy transfer, FRET). Accordingly, a number of requirements also apply regarding the light collector. In particular, the resonance condition imposes certain restrictions on the spectral characteristics of an energy donor and an energy acceptor. Taking into account the fact that the spectral properties of HC components largely depend on their structural properties, which can change during the complex formation, we obtain a complicated multicomponent system whose design optimization is among the most consequential issues in applied biophysics.

Luminescent nanoparticles (LNPs) are currently the most commonly used as light collectors for PS molecules [4]. Such nanoparticles can be applied simultaneously as an antenna, a diagnostic marker, and a platform for targeted drug delivery. A sufficient number of reviews have focused on the latter two aspects of nanoparticle use [5, 6, 7], whereas the fundamental problems of using nanoparticles as a light collector receive less attention [8, 9, 10].

Upconversion [11, 12], silicon [13], and carbon [14, 15] nanoparticles are the LNPs most widely used in photobiology. Furthermore, a large number of studies have been devoted to the application of semiconductor nanoparticles (quantum dots, QDs) as energy donors for PS, although their biocompatibility still remains disputable [16]. Nevertheless, the question regarding the relationship between the spectral and structural properties of QDs is the one that has been resolved most satisfactorily, making it possible to study the energy transfer in HCs in detail.

This review considers the features of the design of HCs based on QDs and PSs with allowance for the complex formation mechanism, the stoichiometry of the complex, the structure of HC components, as well as the influence of these parameters on the efficiency of energy transfer and ROS generation in the complexes. We found out that the photodynamic properties of PS decrease with a rising ratio of the components of the PS : LNP complex because of its high local concentration on the nanoparticle surface even through energy transfer efficiency is enhanced. Enhancement of the luminescent properties of QDs due to protective shells can reduce the efficiency of energy transfer in HC, as the distance between the energy donor and the acceptor increases. Based on the correlations obtained, a technique allowing one to synthesize highly efficient HCs has been proposed; the aim of this technique is to maximize the generation of reactive oxygen species by the photosensitizer within a HC. The conclusions drawn in this review largely apply to HCs based on all other types of LNPs.

## 1. COMPONENTS OF THE HYBRID COMPLEX

**1.1. Second-generation tetrapyrrolic photosensitizers**

A photosensitizer that is highly efficient in terms of ROS yield is supposed to boast the following characteristics. First, the energy of its triplet state must be sufficient to enable a photodynamic reaction with molecular oxygen; the selection is performed to increase the yield of the triplet state and its lifetime. Second, the PS is supposed to exist in a monomeric state, since PS aggregates do not generate ROS as efficiently. Third, the PS should have a high absorption capacity, preferably within the “optical window” of biological tissues.

It is obvious that the photodynamic properties depend on the structure of a PS molecule. We will consider the relationship between the structural and photophysical properties of PSs using tetrapyrrole dyes, the most common second-generation PSs, as an example.

**Fig. 1:**
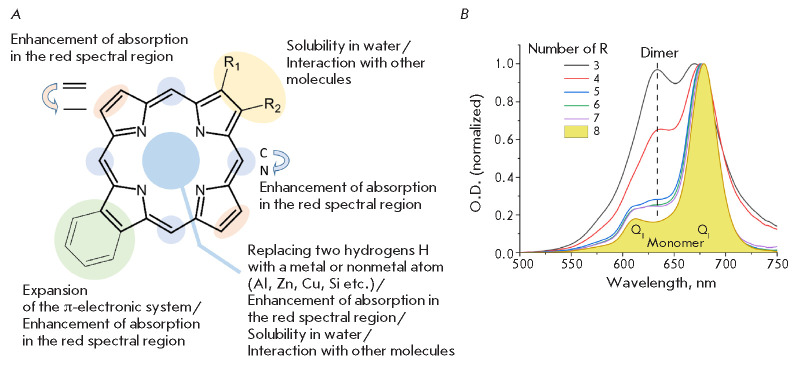
(*A*) – The structure of a porphin molecule and possible ways for its modification. (*B*) – The absorption spectra of zinc phthalocyanines modified with different numbers of choline groups R

Porphin is the simplest dye of the tetrapyrrole series. The absorption spectrum of porphin contains an intense Soret band at the boundary between the UV and visible regions, as well as four low-intensity narrow bands in the visible region (QI–QIV; numbering starts at longer wavelengths). There are several main ways to modify the structure of a porphin molecule, making it possible to obtain PS that exhibit high photodynamic activity (*Fig. 1A*):

(A) sequential hydrogenation of two double bonds, which are not formally included in the conjugated system, shifts the QI band to the long-wavelength spectral region (a bathochromic shift) and increases its intensity by more than an order of magnitude. Hydrogenation gives rise to the classes of dihydroporphyrins (chlorins) and tetrahydroporphyrins (bacteriochlorins);

(B) replacement of carbon in the methine CH groups with a nitrogen atom (tetrazaporphyrins) or incorporation of benzene rings in the macrocycle of a dye molecule (tetrabenzoporphyrins) increases the intensity of the QI and QIII bands, as well as causes their bathochromic shift. The strongest effect is obtained when these two approaches are combined, i.e., in the classes of tetrazatetrabenzoporphyrins or phthalocyanines (Pcs); and

(C) coordination of various elements by the macrocycle of a porphin molecule due to the lone electron pairs of the central nitrogen atoms. For porphyrins, complexes with divalent metals are the most typical. Formation of a metal complex leads to the degeneration of four absorption bands in the visible spectral region to leave two bands whose intensity is significantly increased. This situation is typical of all porphyrin dyes containing no hydrogenated pyrrole rings. When a metal atom is incorporated into the Pc macrocycle, insignificant bathochromic shifts of the QI and QII bands are observed and the magnitude of the bathochromic shift increases as the atomic number of the metal increases [17].

Modification of the porphyrin structure also changes the characteristics of the excited triplet state. Thus, the yield of the excited triplet state slightly decreases as one proceeds from porphyrins to chlorins [18]. Heavy and paramagnetic metal atoms within the Pc increase the probability of a singlet–triplet transition; therefore, such Pcs are characterized by a high yield of the excited triplet state [19]. In addition, the probability of nonradiative deactivation to the ground state increases due to the involvement of the *d*-shells of the metal in the conjugation system [20]. The relationship between the constants of these processes depends on the nature of the metal and side substituents [21].

The solubility of a tetrapyrrolic PS in water is achieved by incorporating side substituents at the macrocycle periphery. These side substituents are usually low-molecular-weight ligands imparting polarity and/or charge to a molecule [22]. The maximum number of side substituents that can be inserted into a tetrapyrrole molecule is determined by the number of binding sites on pyrrole (or benzene in the case of benzoporphyrins) rings and is equal to eight for both the ortho- and meta-substitution [23]. For silicon PS (or PS complexes with trivalent metals), insertion of axial ligands is available [24]. Side substituents significantly affect the optical and photophysical properties of PS [17, 25, 26]. A wide range of substituents with specific properties, as well as the possibility of varying the degree of substitution, allow one to create substituted PSs for various fields of industry (catalysts, sensors, and solar cells) or medicine.

Although chemical modification of PS molecules makes them more water-soluble, the hydrophobic nature of the macrocycle determines the probability of aggregation of these molecules in aqueous solutions. Several types of tetrapyrrole aggregates have been proven to exist [27]. H-type (oligomeric) and D-type (dimeric) aggregates have a narrow absorption band in the visible region, which is shifted to the blue spectral region compared to the absorption band of monomeric form (*Fig. 1B*). Tetrapyrrole molecules in such aggregates form a “sandwich” structure; the aggregates do not fluoresce, since the excited state is nonradiatively deactivated due to intramolecular conversion. J-type (polymeric) aggregates have a wide absorption band shifted to the red spectral region, compared to the absorption band of the monomeric form; the aggregates are formed by PS molecules interacting with the edge parts. Porphyrin molecules can simultaneously exist in both forms (the monomer/aggregate equilibrium) of all types of aggregates; transitions between these states are also possible [28, 29, 30]. Aggregation can be caused by variation of a number of ambient parameters (pH and ionic strength of a solution) [31, 32] or an increase in PS concentration [33]. It can also be initiated by the formation of a complex between tetrapyrroles and molecules of a different nature [34]. The probability of aggregation also depends on the presence and nature of the central metal atom in the PS macrocycle.

**1.2. Colloidal quantum dots**

**Fig. 2:**
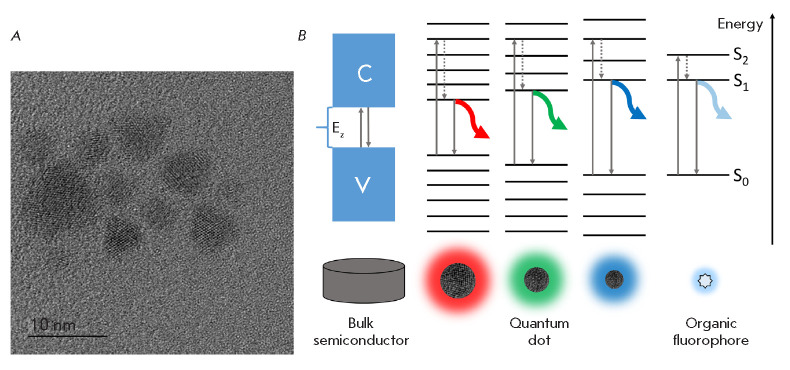
(*A*) – An electron micrograph of CdSe/ZnS nanoparticles. (*B*) – The energy spectra of a bulk and nanosized semiconductor vs. the energy spectrum of an organic fluorophore. C – the conduction band; V – the valence band; Ez – the forbidden band; and S0, S1 and S2 – the ground, first and second excited electronic levels, respectively. Vertical arrows indicate the electronic transitions; dashed arrows indicate the transitions to the lower excited levels accompanied by thermal energy dissipation

Quantum dots simultaneously have the physical and chemical properties of molecules and the optoelectronic properties of semiconductors. A QD is a luminescent semiconductor nanocrystal whose characteristic dimensions lie in the range of 3–10 nm (*Fig. 2A*). It is known that the properties of nanomaterials qualitatively differ from those of a bulk analog [35] because of the quantum size effects. If the size of an object does not exceed the Bohr radius of the exciton, typical of a given material, the charge carrier inside the object appears in a three-dimensional potential well [36]. This leads to a modification of the energy spectrum (*Fig. 2B*). The classical spectrum of a semiconductor with a valence band, a forbidden band, and a conduction band is transformed into a set of discrete energy levels with a characteristic gap *h*2/8*π*2*mr*2, where *h* is the Planck constant, *m *is the effective mass of the charge carrier, and *r *is the QD radius. Electron transitions are possible between these levels, accompanied by absorption or emission of a quantum of light in the visible wavelength range.

Due to the absorbed energy, the electron is transferred to a high-energy level, so that an exciton (an electron–“hole” pair) is formed in the QD crystal. Deactivation of the excited state is performed by exciton recombination accompanied by the emission of excess energy as a light quantum.

Since the gap between the energy levels of QDs depends on particle size, the luminescence spectrum of QDs undergoes a bathochromic shift when the crystal radius is increased. Thus, by varying the crystal size, one can choose QDs with the required spectral properties for specific research problems.

Quantum dots absorb light in a wide wavelength range with molar extinction coefficients of ~10^5^-10^6^ L/mol•Ecm. This fact has spurred a keen interest in QDs as promising luminescent labels for biological research. However, for a successful application of QD in biology, two significant disadvantages of QDs (the low luminescence quantum yield (*φ*) and hydrophobicity of semiconductor material) need to be overcome.

The main reason for the low *φ *values is the crystal lattice defects on the nanocrystal surface, which act as trap states for the charge carrier [36]. The charge carrier localized in such a trap prevents radiative recombination of the exciton. A QD is said to have passed into the so-called “off” state, which can be up to 100 s for an individual crystal [37].

The number of the defects on the QD surface was reduced for the first time in 1990 by coating a CdSe nanocrystal with a protective ZnS shell [38]. We will further refer to the luminescent central part of a multilayer QD as its core. Zinc sulfide is also a semiconductor, but with a wider gap, which creates a potential barrier for the charge carrier and pushes exciton to localize in the QD core. In addition, the protective shell is a physical barrier between the QD core and the environment, making the optical properties of the QD less sensitive to chemical reactions on its surface. By 1996, the development of methods for coating the QD core with a protective shell had given rise to samples of relatively monodisperse nanocrystals with *φ *~ 50% [39]. The modern methods used to synthesize QDs yield nanocrystal samples with a *φ *~ 80–90% [40]. It should be noted that the *φ *value depends nonlinearly on the thickness of the protective shell of the QD: the protective shell consisting of more than three ZnS layers was shown to quench the luminescence of the QD with a CdSe core [41]. It is believed that the probability of formation of intrinsic defects increases with the number of atomic layers in the shell [42].

Furthermore, the use of QDs in biological research involves the transfer of hydrophobic nanocrystals to the aqueous phase. Substitution chemistry methods are usually used for this purpose: the precursor molecules covering QDs during their synthesis are replaced with amphiphilic ligands with the target properties.

Any molecules containing nucleophilic groups can be adsorbed on the nanocrystal surface. The organic shell can be multilayered: an amphiphilic polymer is additionally adsorbed onto a layer of low-molecular-weight hydrophobic ligands, which is responsible for the surface properties of QDs. In addition to water solubility, the organic shell largely ensures passivation of crystal lattice defects [43]. However, organic ligands cannot cover the entire surface of QDs; therefore, some crystal lattice defects persist [36]. In addition, ligands can give rise to new energy levels: thiols are known to quench the luminescence of CdSe QDs due to the emergence of an energy level superjacent to the first excited level of QDs [36].

The typical lifetime values of QD luminescence are 5–20 ns, being quite sufficient for efficient energy transfer. The kinetics of QD luminescence decay are characterized by two or three time components. There currently is no clear understanding of the reasons for the complexity of the decay kinetics of QD luminescence [35]. The most common hypothesis associates each time component with emissions from a specific energy state. This is evidenced by the complex structure of the exciton absorption peak of QDs [44]. In the simplest case (biexponential decay curve), the fast component corresponds to the radiative recombination of an exciton, while the slower one corresponds to the radiation mediated by crystal lattice defects [45, 46]. In this model, the QD luminescence spectrum consists of two overlapping bands, which sometimes cannot be separated. The contribution of the slow component declines with decreasing temperature [45] and luminescence quantum yield [40]. In this case, the luminescence decay curves of ideal QDs without defects would be monoexponential; indeed, only one time component was found in some QD samples [37, 47]. The more differentiated the defects in crystals are (especially in QDs with a core/shell structure), the more time components in the luminescence decay curves there are [39].

Particle size nonuniformity can be an alternative reason for the emergence of several time components in the decay curves of QD luminescence. Increasing the QD size not only leads to a bathochromic shift in the luminescence spectrum, but also causes a corresponding shift of the exciton band in the absorption spectrum [48], reduces the luminescence lifetime [49], and causes nonlinear changes in the quantum yield *φ *[50]. Consequently, a broadened luminescence spectrum will be observed for a sample containing several fractions of QDs of different sizes. Such a spectrum is a superposition of the spectra from different fractions of QDs, which have their own quantum yield and luminescence lifetime values. The average value weighted over all the components is typically used as the QD luminescence lifetime because of the complexity of interpreting the time components.

## 2. THE COMPLEXATION STRATEGIES

**Fig. 3:**
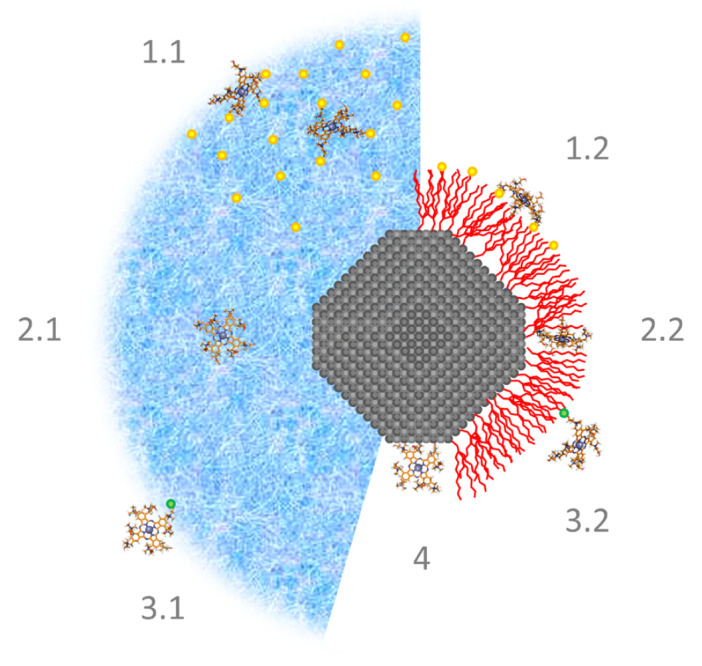
The most common methods used to create a quantum dot–photosensitizer hybrid complex. 1.1–1.2 is the electrostatic interaction; 2.1–2.2 are absorption and adsorption, respectively; 3.1–3.2 are the covalent interactions; 4 corresponds to coordination. The nanocrystal core of QDs is shown in gray; the polymer shell is shown in blue; and the shell of low-molecular-weight ligands is shown in red. The orange dot indicates the charged functional groups on the polymer/ligand; the green dot shows the covalent bond

The following types of interactions make it possible to create hybrid LNP–PS complexes in aqueous solutions: electrostatic or covalent ones, or a group of interactions combined under the concept of sorption (*Fig. 3*). The spectral properties of PS change as bonds of any of these types form. The properties of LNPs change extremely rarely and are not associated with the HC formation process [51, 52, 53].

**2.1. Electrostatic interaction**

HCs are often formed by mixing the aqueous solutions of LNP and PS due to the electrostatic attraction of oppositely charged components (*Fig. 3, 1.1–1.2*). In this case, changes in the spectral properties of PS should be determined by electron density perturbation and may differ depending on the nature and the stoichiometric ratio of HC components. Information on the following complexation effects is available:

(1) a bathochromic shift in the absorption and/or fluorescence spectra of PS [54, 55, 56, 57, 58, 59];

(2) a hypsochromic shift in the absorption and fluorescence spectra of PS [52, 60, 61, 62];

(3) hypochromism [52, 56, 57, 60];

(4) a reduced quantum yield of the PS fluorescence [60, 62];

(5) an increased [60, 63] quantum yield of the triplet state of PS; and

(6) an increased [60, 62] lifetime of the triplet state of PS.

The increased yield of the triplet states of PS is usually attributed to the so-called “heavy-atom effect.” According to it, the probability of intramolecular conversion of PS to the triplet state increases in the presence of heavy metal atoms (Cd, Te), which also reduces the quantum yield of PS fluorescence. In some cases, the cadmium ion from QD can be incorporated into the macrocycle of metal-free PS upon HC formation [62, 64]. It was noted [65] that the magnitude of the changes in the optical properties of PS increases with the size of the QD crystal. The presence of the ZnS protective shell is expected to reduce the effect of the heavy metal atoms in the QD core on the properties of PS.

**2.2. Nonspecific sorption**

HCs formed due to the electrostatic attraction of oppositely charged nanoparticles and photosensitizers do not require special preparation protocols and are quite stable. However, it was noted that mixing of likecharged components also leads to the formation of HC in some cases [66, 67, 68, 69, 70]. Consequently, the self-assembly of HCs can involve interactions other than electrostatic ones, which we will further combine under the term “sorption”.

Depending on the structure of the organic shell of QD, there can be two variants of PS sorption. If the QD surface is coated with a layer of low-molecular- weight ligands, then PS molecules are incorporated into this monolayer due to peripheral [64] or axial [71, 72, 73] hydrophobic substituents. In this case, we talk about surface binding (*adsorption*, *Fig. 3, 2.2*). This kind of interaction weakens with increasing branching of the substituent [73]. Interestingly, the energy transfer efficiency increases with the substituent length as a result of stronger interaction, but then it decreases if the substituent length starts to exceed the length of the low-molecular-weight ligand on the QD surface [72].

**Fig. 4:**
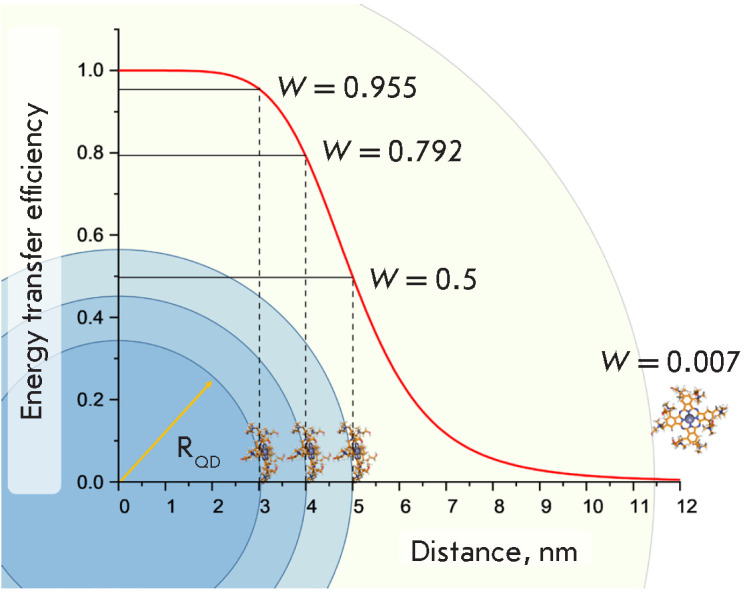
Dependence of the energy transfer efficiency *W* in HC on the size *R*_QD_ of the QD core (here, we assume that the coordination bond forms directly between the PS molecule and the QD crystal). The Forster radius (*R*_0_) was chosen to be equal to 5 nm. An example of HC with a photosensitizer covalently attached to the QD polymer shell (the total QD radius being 11.5 nm) is shown

Direct interaction between a PS molecule and a QD crystal is a special case of adsorption (*Fig. 3*, *Fig. 4*). The formation of a coordination bond between the tertiary nitrogen atom of the PS molecule and the atoms of the CdSe/ZnS QD crystal lattice in toluene can be considered proven [74, 75, 76, 77, 78]. In this case, a close contact is required between the PS and the QD crystal, which can be hindered by the outer organic shell of the nanoparticle. Meanwhile, the formation of a coordination bond should not be accompanied by the obligatory displacement of organic ligands by the PS molecule, since adsorption can occur on the ligand-free areas of the nanoparticle surface. A porphyrin molecule can obviously be adsorbed onto QDs both by the plane of the macrocycle involving all the side pyridyl rings and by its edge involving one or two pyridyl substituents. This is evidenced by the increased efficiency of energy transfer W in HC as the number of pyridyl substituents in the porphyrin molecule rises from 1 to 4, but the value of W is comparable for monopyridyl porphyrin and bipyridyl porphyrin with an opposite arrangement of pyridyl rings.

A hypsochromic shift in the fluorescence spectrum of porphyrin and an increase in its fluorescence lifetime were observed during the formation of HC [79]. According to Zenkevich et al. [79], the amplitude of the effects decreased with increasing porphyrin concentration in solution due to the rise in the proportion of porphyrin molecules not associated with QDs. In addition, during the formation of HC, a bathochromic shift of the Soret band was observed, which is possibly caused by changes in the structure of the π-system of electrons upon coordination of the pyridyl nitrogen atom to the QD zinc atom, or by a higher dielectric constant of the medium near the nanoparticle surface as compared to the toluene solution.

The feasibility of coordination interaction has been proposed to explain the formation of HC between a negatively charged CdTe quantum dot (coated with 3-mercaptopropionic acid) and aluminum tetrasulfophthalocyanine, which is also negatively charged [80]. It is assumed that the PS molecule is coordinated to the QD carboxyl group by an aluminum atom. The idea was extended to CdTe QDs coated with thioglycolic acid [81, 82].

The complexes of PS with LNPs coated with a polymer shell are of particular interest [70, 83, 84, 85, 86, 87, 88, 89, 90]. It is believed that the PS molecules in such complexes can be incorporated into the bulk of the polymer; therefore, we talk about *absorption *in this case (*Fig. 3*, 2.1). This conclusion was drawn from the fact that the hydrodynamic radius of an LNP (together with the polymer shell) exceeds the distance between the donor and the acceptor required for efficient energy transfer via the FRET mechanism that is actually observed in these systems.

During the formation of HC through sorption, multidirectional changes in the spectral properties of PS were noted, depending on the type of PS molecule [78, 85, 87, 89, 91, 92]; there could also be no changes at all because of the adsorption occurring when incorporation was minimal [64]. Aggregation of PS molecules can be observed during sorption [93].

The triplet yield of PS typically increases upon sorption on LNP [67, 92]; however, some opposite results have also been obtained [68]: thus, a reduced lifetime of the triplet state of PS was observed in [91]. This could have been due to the fact that when a PS molecule is incorporated into the organic shell of a QD, the probability of quenching of the PS triplet state by oxygen decreases [78].

**2.3. Covalent binding**

The complexes between nanoparticles and PS formed via covalent interaction have a number of advantages over HCs stabilized by other types of interactions (*Fig. 3, 3.1–3.2*). First, the interaction occurs between specific functional groups of PS and the organic shell of QDs; therefore, exact localization of PS in HC is known. This makes it possible to predict some of the photophysical properties of HCs. Second, this HC potentially remains more stable in the presence of biological objects and environments. Therefore, there is a keen interest in covalently stabilized conjugates of PSs and nanoparticles [53, 65, 94, 95, 96, 97, 98, 99, 100].

Formation of a covalent bond can be easily monitored by the emergence of corresponding lines in the Raman spectra or absorption spectra in the IR region [65, 94, 96]. Meanwhile, it is difficult to control the PS : LNP ratio in the end product when routine crosslinking methods are used. In addition, when crosslinking is performed through amino and carboxyl groups, HCs of the electrostatically interacting components can form, which is difficult to prevent. For these reasons, a number of studies have failed to compare the properties of covalently crosslinked and electrostatically stabilized HCs based on the same components [94, 96, 98].

An even more important problem is that the linker that forms between the PS molecule and the LNP surface increases the distance between them. This fact negatively affects the energy transfer efficiency, which rapidly decreases with increasing distance between the energy donor and acceptor. *Figure 3* shows that the influence of this effect can be critical when a polymer shell is used.

In most studies, changes in the spectral characteristics of PS during HC formation are identical for the covalent and electrostatic binding methods: a hypsochromic shift in the absorption spectrum of PS and hypochromism [94, 95, 97] or a bathochromic shift in the absorption spectrum of PS and hypochromism [98]. There were no changes in the spectral properties of PSs during the formation of a covalent bond [96].

## 3. DESIGN OPTIMIZATION FOR HYBRID COMPLEXES

Designing hybrid complexes based on LNPs implies that the efficiency of ROS generation by a photosensitizer upon excitation is increased in the spectral regions where the PS itself has a low absorption capacity. Since such an enhancement of the photodynamic properties of PS is achieved due to nonradiative energy transfer, optimization of the HC design is primarily associated with the optimization of energy transfer via the FRET mechanism. However, it should be noted that a set of properties of HC promoting efficient energy transfer may, generally speaking, not coincide with the set of properties of HC that enhances the photodynamic activity of PS in HC. For this reason, we will consider these two aspects of HC optimization separately.

**3.1. Energy transfer efficiency**

Since energy transfer increases the deactivation rate of the excited state of an energy donor, the degree of quenching of LNP luminescence is the main criterion in a quantitative assessment of the transfer efficiency.

Let us consider the simplest quantum dot–tetrapyrrolic PS system stabilized via coordination of the PS to the nanocrystal surface. The subject of optimization will be energy transfer, which contributes to the increase in the absorption capacity of PS in the bluegreen spectral region. According to the nonradiative resonance energy transfer theory, the efficiency of this process (*W*) can be increased by

(A) increasing the overlap integral (*J*) of the LNP luminescence spectrum and PS absorption spectrum;

(B) increasing the quantum yield of LNP luminescence;

(C) decreasing the LNP–PS distance;

(D) increasing the molar extinction coefficient of a PS molecule; or

(E) increasing the PS : LNP stoichiometric ratio.

The *J *value can be increased by shifting the QD luminescence spectrum to longer wavelengths, closer to the absorption spectrum of the PS. Since the position of the luminescence spectrum of QDs is easily specified during their synthesis, a QD providing the maximum* J *value can be easily selected when the position of the PS absorption spectrum is fixed. However, the bathochromic shift in the QD luminescence spectrum occurs due to a rise in the particle size, which increases the QD–PS distance and reduces the energy transfer efficiency *W* (*Fig. 4*). This is typically accompanied by a reduction in the quantum yield of QD luminescence, which should also negatively affect the* W *value. Although the quantum yield of QD luminescence can be increased by growing a protective shell from a wider-gap semiconductor, such a modification will not only increase the luminescence yield, but also additionally increase the crystal size and, accordingly, increase the donor–acceptor distance. An alternative way is to choose materials for the crystal lattice of the QD core. On the one hand, an organic shell on the QD core protects the crystal surface against solvent molecules; therefore, the QD luminescence yield is expected to increase. On the other hand, additional defects may form on the crystal surface depending on the nature of the molecules comprising the organic shell, and the quantum yield of the QD luminescence will decrease.

It is possible to increase the *J *value due to the hypsochromic shift in the absorption spectrum of PS, since QDs of a smaller size can be used to create HCs in this case. Indeed, a smaller QD size will increase the quantum yield of QD luminescence and reduce the QD–PS distance, which will eventually increase the *W *value. However, applying such a strategy means that the red spectral region will not be used for ROS generation. In addition, if the spectra are ultimately shifted to the blue region, there is no need to use QDs, since many metal-free PSs absorb blue light perfectly due to the Soret band.

Therefore, complex QD-based systems have a number of parameters that cannot be optimized simultaneously due to their mutually exclusive influence on each other. Consequently, the highest energy transfer efficiency can be achieved only through compromise values of the PS and QD parameters.

Variation of only two parameters unambiguously increases the *W *value: increasing the molar extinction coefficient of PS and the PS : LNP stoichiometric ratio.

The molar extinction coefficient of PS in the visible region is usually increased by inserting a metal atom into the macrocycle. Since the formation of a metal complex significantly increases the lifetime of the triplet state of PS, this additionally enhances the photodynamic activity of the PS. Alternative ways for increasing the molar extinction coefficient of PS are to replace carbon with nitrogen in the methine bridges of the macrocycle, increase the macrocyclic aromaticity due to benzene rings, and hydrogenate double bonds. These ways also lead to an additional bathochromic shift in the absorption spectrum of PS. Consequently, it is necessary to additionally shift the luminescence spectrum of QD to longer wavelengths to preserve the maximum value of the overlap integral *J*. The effects caused by such a displacement can reduce the efficiency of energy transfer in HC.

The PS : LNP stoichiometric ratio can be increased to a certain limiting value that depends on the complexation method. If HC is formed by covalent crosslinking, then [PS : LNP]_max_ is determined by the number of functional groups on the organic shell of the QD (i.e., their density and surface area of the QD). If HC is stabilized via electrostatic interactions, the [PS : LNP]_max_ is determined by the number of charged groups on the organic shell of QD, as well as the number of charged groups on the PS molecule. There is ambiguity here: the more charges there are on the PS, the stronger the interaction is, but fewer PS molecules will bind to the QD surface.

If HC is stabilized trough sorption interactions, the [PS : LNP]_max_ is determined by the LNP surface area, as well as by the hydrophilic–hydrophobic balance of the PS molecule. For a bulk polymer shell of a nanoparticle, [PS : LNP]_max_ will be much higher than that when a monolayer of low-molecular-weight ligands is used. However, additional PS molecules will be located far enough from the QD center so that the efficiency of energy transfer to these PS molecules should be minimal. *Figure 4* shows the situation where PS is covalently bound to the polymer shell of QD. One can see that for a total nanoparticle radius of 11.5 nm and Forster radius R0 = 5 nm, the efficiency of energy transfer to a given PS molecule will be no more than 0.7%.

In theory, the increased factor *χ*2 describing the mutual orientation of the transition dipole moments of the donor and acceptor can increase the energy transfer efficiency. The *χ*2 values can vary from 0 to 4. In solutions,* χ*2 is taken equal to 2/3 due to rotational diffusion and random orientation of the molecules. This is also used in the case of HCs, since most QDs do not have luminescence anisotropy. Nevertheless, in the general case, the orientation of transition dipole moments in the HC can be nonrandom. It is assumed that studies focusing on the anisotropy of the PS and LNPs fluorescence would potentially help estimate the possible mutual orientations of the transition dipole moments and thereby refine the *χ*2 value [101].

**3.2. Photodynamic properties of a photosensitizer**

A successful energy transfer event causes a transition of the PS molecule to an excited state. Energy transfer can increase the ROS yield or increase the intensity of PS fluorescence. Increased absorption capacity of a PS manifesting itself as an increase in the intensity of its sensitized fluorescence can be used to calculate the energy transfer efficiency *W *[58, 75, 82]. However, it is considered more correct to use the spectral characteristics of the energy donor to calculate the *W *value, since enhancement of the photodynamic properties of PS in HC strongly depends on the PS : LNP stoichiometric ratio.

**Fig. 5:**
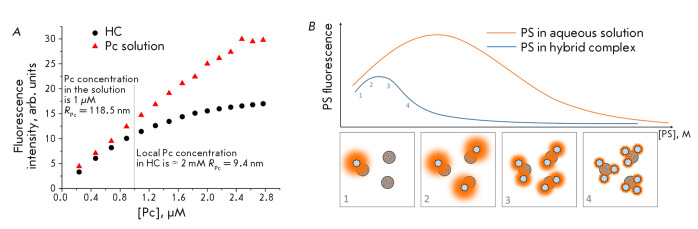
(*A*) – The concentration dependence of the fluorescence intensity of polycationic aluminum Pc in an aqueous solution and in an electrostatically stabilized hybrid complex with a polymer-coated QD. *R*Pc is the average distance between two Pc molecules in the medium (water in the case of a one-component Pc solution and the QD polymer shell in the case of a HC solution). The fluorescence excitation wavelength is 655 nm. (*B*) – The concentration dependence of the PS fluorescence intensity in water and in HC (LNP concentration being constant). (1–4) the schematic representation of HCs with a different stoichiometry and the fluorescence intensity of PS in such HCs

It is known that as the PS concentration in a dilute solution rises, its fluorescence intensity increases linearly in the initial period of time; however, it reaches a plateau and then decreases in sufficiently concentrated solutions (*Fig. 5*) [102]. This effect can be called “self-quenching of PS fluorescence”. Self-quenching of the PS fluorescence can be caused by PS aggregation and the inner filter effects. PS aggregation was discussed in section 1.1. The inner filter effects consist in the shielding of the exciting light by layers of the PS solution, which lie closer to the front cell wall (a), and reabsorption of PS fluorescence (b). The latter is possible, since tetrapyrrolic PSs have a small Stokes shift (~ 10 nm) so that the absorption and fluorescence spectra of PSs largely overlap. In addition to the nonlinear dependence of PS fluorescence intensity on its concentration, this phenomenon leads to a bathochromic shift in the fluorescence spectrum of PS and increases the measured fluorescence lifetime of the PS [103].

Quenching of PS fluorescence in the presence of nanoparticles is common [55, 104, 105, 106]. The concentration dependence of the fluorescence intensity of PS in HCs with semiconductor nanoparticles is also nonlinear [59, 70, 71, 87]; however, self-quenching starts at a much lower PS concentration compared to the PS in a single-component solution (*Fig. 5A*). Indeed, the maximum PS : LNP ratio in HC can exceed 1000, so the local PS concentration during complex formation can be as high as several mM [90].

An increasing PS : LNP ratio may result in the aggregation of PS in the organic shell of the LNP. This effect is observed in any type of interaction between PS and LNP, except for covalent crosslinking. Any PS in a solution exists in a state of monomer/aggregate dynamic equilibrium, which can be shifted upon binding to LNP. The probability of this process depends both on the structural properties of the PS molecule (the type of metal atom, the nature and number of peripheral substituents) and on the structural features of the organic shell of the LNP. Thus, we have shown that despite the presence of eight peripheral carboxyl groups, zinc and aluminum Pcs aggregate upon binding to upconversion LNPs coated with a polymer shell containing terminal amino groups; zinc Pcs undergo aggregation at lower concentrations than aluminum Pcs do [107]. In this case, the PS aggregates continue to accept the electronic excitation energy of the LNP and the efficiency of this process may increase due to the greater overlap of the absorption spectrum of the aggregates with the luminescence spectrum of the LNP.

In addition, concentrating PS from the solution onto the LNP surface leads to solution “bleaching” within the region of PS absorption. In this case, the photodynamic activity of PS in HC is further reduced.

Let us imagine that the number of PS molecules on the LNP surface can increase infinitely without an increase in the average PS–LNP distance that is equal to the Forster radius R0. According to Forster’s theory, at PS : LNP = x = 1, the energy transfer efficiency *W *is 50% at a distance R0. When x = 10, *W *= 91%; at x = 100,* W *= 99%; and at x = 1000, *W *= 99.9%. It is clear that the highest increase in the *W *value is observed as the PS : LNP ratio rises from 1 to 10, which is much less than the characteristic [PS : LNP]_max_ values are. It is fair to say that the absolute energy transfer efficiency* W *increases with a rising number of PS molecules in HC, while the energy transfer efficiency *W *for every separate PS molecule decreases.

Consequently, the more PS molecules there are in a complex with LNP, the less additional energy each of them receives, and, therefore, the enhancement of photodynamic properties of PS tends to zero. The photodynamic activity of PS in the HC at large PS : LNP ratios turns out to be lower than the activity of free PS due to self-quenching effects.

Finally, the use of some types of LNP shells can lead to the fact that ROS formed in a reaction between PS and molecular oxygen inside the organic shell of LNP cannot effectively damage the targets in the solution surrounding HC, since diffusion in the LNP shell is hindered. In this case, the most likely target of oxidation will be the PS molecule itself. Indeed, in electrostatically stabilized HCs based on aluminum phthalocyanines and QDs coated with a polymer shell, we observed rapid bleaching of the dye both under selective illumination of Pc and upon excitation of QD, followed by energy transfer [108]. As a result, the measured concentration of ROS is lower than the actual one. Nevertheless, the calculated ROS concentration corresponds to the effective concentration of ROS capable of exhibiting photodynamic activity outside the hybrid complex.

Therefore, the increased energy transfer efficiency in HCs due to a rise in the PS : LNP value contradicts the idea of enhancing the photodynamic activity of PS.

It should be noted that the interaction between PS and LNP can result in electron transfer. This phenomenon is observed quite rarely and is easily detected with strong changes in the spectral properties of PS due to the formation of radical anions and other derivatives [63, 109]. In addition, the electron transfer implies a QD transition to the “off” state, when the model of classical static quenching is appropriate. In this case, the QD luminescence intensity is quenched without a change in its lifetime. Unfortunately, the luminescence lifetime of LNPs has been estimated only in some studies and the absence of such an estimate may lead to a misinterpretation of the experimental results [52, 56].

## CONCLUSIONS AND FUTURE PROSPECTS

**Fig. 6:**
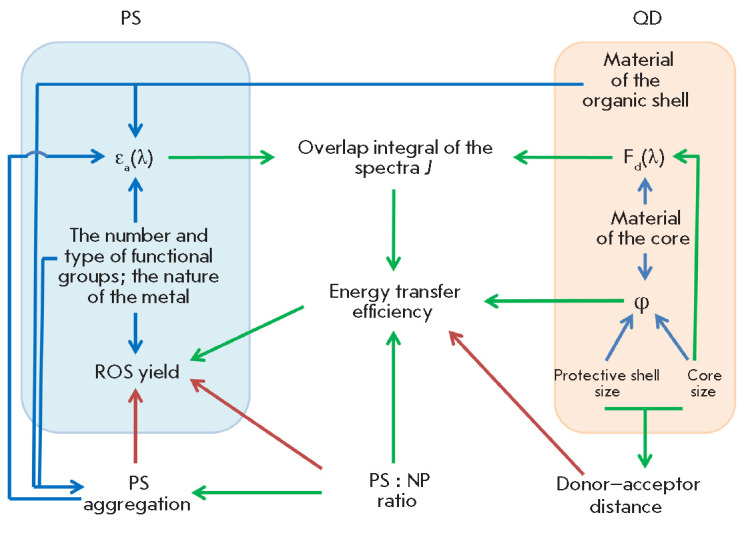
Scheme showing the functional relationships between the structural parameters of PS/QD molecules and their photophysical properties, as well as the effect of these properties on the yield of reactive oxygen species through the parameters of energy transfer via the FRET mechanism. *F_d_*(*λ*) is the luminescence spectrum of QD; *ε_a_*(*λ*) is the absorption spectrum of PS; and *φ *is the luminescence quantum yield. Green arrows denote the positive correlation; red arrows denote the negative correlation; and blue arrows show the nonlinear dependences

All the mentioned functional relationships between the structural and spectral properties of PSs and LNPs, which can affect the efficiency of LNP as a light collector, and an enhancement of the photodynamic activity of PS in HC can be summarized in a single scheme shown in *Fig. 6*. One can see that all the key characteristics of PS and LNPs are interconnected. Therefore, the full set of parameters optimized so as to ensure the highest ROS yield must involve some degree of compromise.

Achieving this compromise is the primary task for PDT on its path to creating third-generation PSs. However, even though an impressive number of studies have been devoted to HCs, the data collected are too fragmentary and heterogeneous, making a global analysis and the selection of the required set of HC characteristics impossible. This would be feasible only by using an integrated approach, when all the connections shown in *Fig. 6* can be identified as quan titative dependences. Since most of these parameters are related to each other by the well-known formulas of the FRET theory, the difficulty arises only at the stage of uncovering the relationship between the structural and photophysical characteristics of the HC components. First of all, this concerns LNPs, since the relationship between the structural and spectral properties of tetrapyrrolic PSs has been studied quite thoroughly.

However, it is not enough to possess information about the properties of each component to optimize the design of the HC. Phenomena such as PS aggregation and static quenching of the luminescence of LNPs (as a result of the formation of nanocrystal surface defects involving PS) can be quantitatively studied only through experiments on HC formation. It should also be noted that electron density perturbation in a PS molecule during the formation of HC (even in the absence of the aforementioned aggregation and quenching effects) has some effect on the photophysical properties of PS and, thus, indirectly affects the energy transfer efficiency and the enhancement of the ROS yield. Failure to take into account any of the parameters described above leads to the following fact: even in the presence of PSs and LNPs with spectral characteristics optimal for FRET, it might not always be possible to obtain HC where enhanced PS fluorescence or the ROS generation rate is observed [87, 98, 104, 105, 106]. This usually leads to a rejection of the FRET mechanism as a model for describing the interactions between a nanoparticle and a PS [51, 56, 91, 93, 110].

It might be possible to find several variants of complexes significantly differing in terms of their set of internal characteristics but having comparable ROS yields (or comparable in terms of the efficiency of using certain spectral regions for ROS generation) by optimizing the HC design. Since the enhancement of the photodynamic characteristics of PS can be achieved only at low PS : LNP values, when the luminescence of the LNP is not completely quenched, the LNP luminescence can be used for diagnostic purposes. Such HCs can obviously be used to solve specific problems of PDT and fluorescence diagnostics depending on the properties of the target object. In this regard, it must be said that we have discussed the trends in optimizing the HC design exclusively with a view to enhancing the ROS yield. In fact, the overall photodynamic activity will depend not only on the absorption capacity of HC and the ROS yield, but also on the efficiency of interaction between HC and cells, the internalization mechanism, and the stability of HC in the presence of blood components when an HC-based drug is administered to a living being. It is highly likely that the approaches to optimizing HC for increasing the efficiency of targeted delivery will significantly affect the final set of HC parameters. Therefore, the scheme shown in *Fig. 6* should be expanded with allowance for all the aspects of the functional activity of HC as a third-generation photosensitizer. Building a complete scheme of this kind will allow one to take the prospects for using HC with energy transfer in PDT to a fundamentally new level and is, therefore, the main objective of modern medical biophysics.

## Abbreviations

HC: hybrid complex
LNP: luminescent nanoparticle
Pc: phthalocyanine
PDT: photodynamic therapy
PS: photosensitizer
QD: semiconductor quantum dot
ROS: reactive oxygen species
